# Antibiotic resistance and burden of foodborne diseases in developing countries

**DOI:** 10.4155/fsoa-2016-0023

**Published:** 2016-09-01

**Authors:** Olumide A Odeyemi, Norrakiah Abdullah Sani

**Affiliations:** 1 Aquatic Microbiology Laboratory, Ecology & Biodiversity Centre, Institute for Marine & Antarctic Studies (IMAS), University of Tasmania, Launceston, Australia; 2 Food Safety Research Group, School of Food Technology & Chemical Sciences, Faculty of Science & Technology, National University of Malaysia, UKM Bangi, Selangor, Malaysia

**Keywords:** antimicrobial resistance, food safety, foodborne pathogens

**Figure 1.**
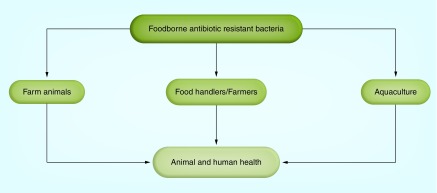
Spread of foodborne antibiotic resistant bacteria in the environment.

The emergence and re-emergence of antibiotic-resistant foodborne bacteria in recent times calls for concerted efforts, especially in developing countries. Children and immunocompromised individuals suffer most from these pathogens. This article highlights burden of antibiotic-resistant foodborne diseases (FBDs) and possible solutions, focusing on developing countries.

## Foodborne diseases

Infectious, FBDs acquired from contaminated cooked, raw, processed or unprocessed food are major courses of mortality and morbidity globally due to their wide and rapid spread [1,2]. This article aims to highlight the burden and causes of FBDs, and reduction measures in developing countries. Due to them being widespread, these diseases are of public health interest worldwide, as all countries suffer incidence of FBDs although the levels differ from country to country [2]. More than 250 FBDs originating from microbial, chemical or parasitic sources have been identified [3].

## Burden of FBDs

The burden of FBDs is global and therefore requires global efforts in terms of collaboration, funding, awareness and commitment from various governments, especially in developing nations, and policy makers. This is because most FBD outbreaks occur at home, restaurants and/or at social functions. Due to the continuous increase in prevalence and the emergence and re-emergence of FBDs, the WHO initiated the Foodborne Disease Burden Epidemiology Reference Group in 2006 to investigate the global burden of FBD. According to a recent report by the WHO, 600 million, or one in ten, people fall ill globally as a result of FBDs with over 91 million people affected in Africa. 700,000 children in one WHO region in southeast Asia die of unsafe food yearly [2]. Diarrhea remains the leading cause of death, especially among children in developing and resource-poor countries. As such, the importance of food safety cannot be overemphasized in developing countries [4]. What is more, a recent report by the WHO stated that FBDs are prevalent in developing countries [5]. Immunocompromised individuals such as children and the elderly are the most vulnerable with 30% of deaths arising from FBDs occurring among children. The public health importance of FBDs, therefore, cannot be overemphasized, owing to the cost effect on individuals, industries, families and the national economy at large [6].

Consumer behaviors, such as food handling and hygiene practices, play a significant role in occurrence of these diseases. Food prepared and/or eaten at home accounts for most of the major reports of FBD outbreaks globally [7,8]. Contamination of food can occur along the food production chain from farm to table. Inadequate cooking, poor water, improper holding temperature and cross contamination have been reported as the major causes of FBD outbreaks in homes [9]. It is therefore imperative that consumers are made aware of the necessity of good personal hygiene and safe food practices so as to prevent outbreaks at home [10]. Various measures have been implemented to reduce incidence of FBDs both in developed and developing countries. However, there has been increased occurrence of emerging and re-emerging FBDs. Among the factors responsible for this is the resistance of foodborne pathogens to antibiotics. This is exemplified by the recently emerging foodborne pathogens *Cronobacter sakazakii*, *Helicobacter pylori*, *Listeria monocytogenes*, *Aeromonas hydrophila*, *Salmonella typhimurium*, *Campylobacter jejuni*, *Vibrio parahaemolyticus*, Verotoxigenic *Escherichia coli* such as Enterohemorrhagic *E. coli* and *Yersinia enterocolitica* [11,12].

## Causes of antibiotic resistance

Antibiotics have been being discovered and used for treatment of infectious disease for over five decades. Abuse of use of antibiotics in agriculture, human and veterinary medicines has resulted in instances of antibiotic resistance [13]. In a recent review, factors such as microbial adaption, international travel, ecosystem, inadequate public health strategies or measures, poor political will, poverty, social unrest, natural disasters and advancement of technology and industry were attributed to resistance to antibiotics [12]. Various mechanisms of antibiotic resistance in foodborne pathogens have also been identified, among which are biofilm formation, enzyme modification, efflux and decreased permeability [14]. In the recent *Lancet* series on antimicrobials, principles and measures of access and excess of antimicrobials were emphasized [15–19]; however, less information was reported on impact of antimicrobials on burden of FBDs in low-income and middle-income countries. The spread of foodborne antibiotic-resistant bacteria in the environment occurs across the food chain from farm to table (Figure 1). Antibiotic-resistant bacteria from the environment can enter farm animal feed and can result from indiscriminate use of antibiotics in animal production and aquaculture. What is more, there could be cross contamination across the food chain with a resultant effect on human and animal health.

Use of antimicrobials is increasing globally either for disease prevention or agricultural purposes, thereby leading to natural selection of antimicrobial-resistant bacteria. Humans are exposed to resistant bacteria through sources such as food products, the environment and food handlers. Among the factors responsible for this occurrence and prevalence are animal husbandry practices, poor food-production processes, inadequate food storage infrastructure, unhygienic food handling, limited resources and poorly enforced regulatory standards [5,20–21]. There could be cross contamination of raw food animals with other food such as vegetables, dairy products and egg if improperly stored. Transposons and plasmids responsible for resistance genes could then be transferred from antibiotic-resistant bacteria found in food [22].

Occurrence and prevalence of bacteria resistant to antibiotics is a major problem in developing countries due to the ease of infection resulting from poor living standards and lack of access to adequate medical treatment. It must be stated that no direct link has been established between poverty and antibiotic-resistant bacteria; however, poverty can result in inability to afford quality medical treatment and purchase of appropriate antibiotics [23]. Similarly, poverty leads to poor living conditions that can enhance FBD outbreaks. Therefore, in developing countries there is need for research collaboration, funding from various stakeholders and capacity building so as to reduce the occurrence and prevalence of foodborne pathogens [24].

## Conclusion

Burden of FBD affects all, most especially children in developing countries [24]. There is a need for studies into the national burden of FBDs and concerted effort on antimicrobial-resistance awareness in developing countries. Developing countries need to implement effective and efficient intervention programs that could help reduce burden of FBDs. Such intervention should include food safety education and awareness, good food handlers’ hygiene and national FBD occurrence surveillance and monitoring programs. Similarly, a comprehensive systematic review of occurrence and prevalence of FBDs and antibiotic resistance in developing countries needs to be carried out as adequate information is lacking, thereby making it difficult to take proactive intervention steps toward prevention of these diseases. There is also need for research scientists in developing countries to investigate occurrence of FBDs in each country and also across borders. This will help providing adequate data and information for FBD outbreaks.
